# MitoRes: a resource of nuclear-encoded mitochondrial genes and their products in Metazoa

**DOI:** 10.1186/1471-2105-7-36

**Published:** 2006-01-24

**Authors:** Domenico Catalano, Flavio Licciulli, Antonio Turi, Giorgio Grillo, Cecilia Saccone, Domenica D'Elia

**Affiliations:** 1 Institute of Biomedical Technologies, CNR, Via Amendola 122/D, 70126 Bari, Italy; 2 Department of Biochemistry and Molecular Biology, University of Bari, Via Orabona 4, 70126 Bari, Italy

## Abstract

**Background:**

Mitochondria are sub-cellular organelles that have a central role in energy production and in other metabolic pathways of all eukaryotic respiring cells. In the last few years, with more and more genomes being sequenced, a huge amount of data has been generated providing an unprecedented opportunity to use the comparative analysis approach in studies of evolution and functional genomics with the aim of shedding light on molecular mechanisms regulating mitochondrial biogenesis and metabolism.

In this context, the problem of the optimal extraction of representative datasets of genomic and proteomic data assumes a crucial importance. Specialised resources for nuclear-encoded mitochondria-related proteins already exist; however, no mitochondrial database is currently available with the same features of MitoRes, which is an update of the MitoNuc database extensively modified in its structure, data sources and graphical interface. It contains data on nuclear-encoded mitochondria-related products for any metazoan species for which this type of data is available and also provides comprehensive sequence datasets (gene, transcript and protein) as well as useful tools for their extraction and export.

**Description:**

MitoRes  consolidates information from publicly external sources and automatically annotates them into a relational database. Additionally, it also clusters proteins on the basis of their sequence similarity and interconnects them with genomic data. The search engine and sequence management tools allow the query/retrieval of the database content and the extraction and export of sequences (gene, transcript, protein) and related sub-sequences (intron, exon, UTR, CDS, signal peptide and gene flanking regions) ready to be used for in silico analysis.

**Conclusion:**

The tool we describe here has been developed to support lab scientists and bioinformaticians alike in the characterization of molecular features and evolution of mitochondrial targeting sequences. The way it provides for the retrieval and extraction of sequences allows the user to overcome the obstacles encountered in the integrative use of different bioinformatic resources and the completeness of the sequence collection allows intra- and interspecies comparison at different biological levels (gene, transcript and protein).

## Background

Mitochondria are sub-cellular organelles which play a central role in many important metabolic pathways and are essential for energy production [1]. The control of mitochondrial biogenesis and function strongly depends on the coordinated activity of both the nuclear and mitochondrial genomes [2] and as a consequence, the molecular mechanisms regulating the mitochondrial transcription, translation, post-transcriptional modification, signalling, import, folding and assembly of the mitochondrial components are processes extremely complicated and still not entirely defined [3]. Part of this effort is the elucidation of transcriptional co-regulation networks, which can be seen as one of the most important levels at which nucleus-mitochondrion network connections emerge [4]. The analysis and comparison of nuclear-encoded mitochondria-related sequences within and between organisms could be of great help for their functional characterization, but they require a sufficient quantity of data and appropriate tools for its selection, extraction and analysis. The mitochondrial databases collecting data on nuclear-encoded mitochondrial components currently available are essentially protein sequence databases such as MitoProteome [5], MitoP2 [6] and the Human Mitochondrial Protein Database (HMPDb) [7]. MitoProteome and HMPDb are databases including only human data while MitoP2 collects mitochondrial protein data on four different organisms: man, mouse, yeasts and neurospora. These databases, even if they may be searched, do not provide transcript and gene sequences and tool for the massive extraction of sequence data. MitoDrome [8], a mitochondrial database developed by our group, differs from the above mentioned databases because it adds the gene and the deduced transcript sequences to protein annotation and provides efficient tools for the retrieval and extraction of sequences but it is restricted to only three dipteran species, *D. melanogaster*, *D. pseudoobscura *and *A. gambiae*.

MitoRes, the database we present here, is a specialized mitochondrial resource which has been developed to complement the other available mitochondrial databases in their biological utility and application. In particular, it tries to fill the void of a comprehensive resource of mitochondria-related sequences and, to this end, it collects and integrates data on gene, transcript and protein sequences of any metazoan species from the most accredited worldwide sources. MitoRes provides efficient tools for the retrieval and massive extraction of any type of nucleus-encoded mitochondria-related sequence and sub-sequence (i.e., gene, gene flanking regions, intron, exon, transcript, UTR, CDS, protein and signal peptide) ready to be used for in silico analysis.

It also makes the intra- and inter- species comparison of the protein sequences collected, and integrates information on protein similarity with genomic data. The integration of the protein and gene data helps users to easily assess the conservation of both the protein sequence and the gene structure when compared with their counterparts in other organisms, thus enabling potential correlations not possible on the basis of the protein similarity alone and facilitating the selection/extraction of the best candidates for further and deeper investigation.

In the following sections we describe the construction and content of MitoRes, its graphical interface and usefulness.

## Construction and content

MitoRes is an interconnected knowledge management system consisting of a relational database, a Web Graphical User Interface (GUI) and a sequence export manager tool. It is derived from the MitoNuc database [9], which has been completely rebuilt and extensively modified to better answer to users' needs in terms of the availability of a comprehensive and non redundant collection of data, as well as flexible access to information and rapid extraction of sequences.

### Data sources and implementation

The MitoRes database has been implemented as a relational database using the freely available MySQL Database Management System (DBMS). Three main interconnected building blocks constitute its information content: protein, transcript and gene modules.

The information content of the database is generated using an automated procedure that is composed of a suite of BioPerl and C programs which retrieve and integrate data from external sources, compare and cluster proteins on the basis of their sequence similarity and populate the database records automatically.

In particular, gene chromosome location, sequence and structural information (exon/intron organization) are extracted from the ENSEMBL genome database [10]. Transcript(s) sequence, polyadenylation recognition signal and location along with transcript function are extracted from the RefSeq database [11] while UTR regions are generated using the UTR database [12] as a reference. Protein sequences, along with information on sub-cellular location, tissue specificity, Enzyme Classification code, signal peptide, gene name and its synonyms (if any) are extracted from the UniProt database [13]. The NCBI Taxonomy database [14] is used as a reference for information on the biological source of the protein and the GO database [15] for protein classification.

**Figure 1:**
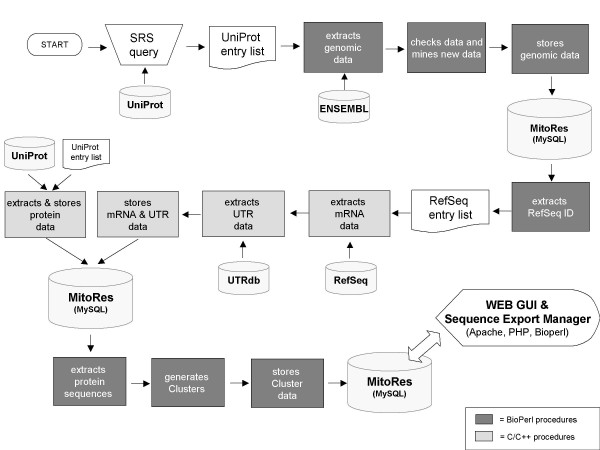
**Flowchart depicting the process used to generate MitoRes**. Dark and clear grey boxes are for BioPerl and C/C++ procedures respectively.

A flowchart describing the whole process is reported in Figure 1. The first step of this procedure is represented by the creation of a list of accession numbers (AC), retrieved from the UniProt database at our SRS (Sequence Retrieval System) web server [16] and relevant to entries containing data on nuclear-encoded mitochondria-related proteins in Metazoa. Completeness and consistency of data retrieved are assured by the use of different combination of search criteria, able to scan the database at different entry levels (i.e., taxonomy, protein description, subcellular location, comments and references) and by automated and manual filtering of the database query results. In particular, the automated filtering is carried out through the use of SRS "query expressions" able to screen the queries results and extract only data of interest. Through this procedure we are able to extract, with a good degree of confidence, all the UniProt entries concerning mitochondrial proteins, discard those reporting incomplete protein sequences (fragment) or proteins which have a mitochondrial genome origin and, on the other hand, detect and keep proteins whose involvement in mitochondrial metabolic networks is not clearly stated in UniProt entry fields (description and subcellular location lines). The results of the SRS "query expressions" are then manually checked for their consistency; the consultation of related literature is also carried out for proteins whose function in mitochondria cannot be deduced from information reported in the UniProt entry.

The list of UniProt ACs obtained through this procedure is then used to upload the MitoRes database using two different programs that act in consecutive steps. The first one, a BioPerl script using the Perl APIs of ENSEMBL, retrieves related genomic information from the ENSEMBL database. The ENSEMBL transcripts extracted, which are only those fully supported by a UniProt link, are checked for their consistency using the blast2seq program [17], to compare their translated sequence against the UniProt sequence, and then used to populate the gene module tables. The second program, a C program using the EMBOSS [18] and UTRdb C libraries, queries the UniProt, UTR and RefSeq databases and retrieves transcript and protein sequences and associated data to populate the other two MitoRes modules, transcript and protein.

The last step of the MitoRes database annotation is represented by the analysis and clustering of the protein sequences. The protein sequences stored in the MitoRes database are extracted and examined for their sequence similarity using an "all-versus-all" pair-wise global alignment and hence clustered in sub-groups (Clusters) on the basis of a threshold sequence similarity value of at least 60%. The entire procedure has been automated by using a Perl script which includes the Strecher EMBOSS programme [19]. The procedure iteratively runs the pair-wise alignment for each protein against the entire collection of sequences and generates a cluster for each iterative step. Data deriving from the protein clustering procedure are automatically uploaded, by means of Perl scripts, into a database table, named Cluster, belonging to the protein module. Clusters take their name from the protein, indicated as "Leader protein" corresponding to the first sequence used by the alignment procedure for comparison with all the others present in the MitoRes collection. From this analysis, currently only about 10% of proteins present in MitoRes do not have counterparts with any other proteins in the database.

### Entry description

The core entity of the MitoRes database is represented by the protein so that each MitoRes entry is generated for each nuclear-encoded mitochondria-related protein reported in the UniProt database. The association of the related nuclear transcript and gene data depend on the availability of relevant annotation across the RefSeq and ENSEMBL databases respectively, thus some entries may be complete whereas others may contain only information on a protein and transcript or only on a protein. Apart from the completeness of the information reported, the general structure of a MitoRes entry is comprehensive of information on gene, transcript and protein.

Each database entry is identified by an Identification (ID) code and by an Accession number (AC) which serves for unambiguous retrieval and citation, from release to release. Gene and transcript sequences are graphically represented through the dynamic construction of their physical maps which are descriptive of their structure, sequence orientation and genomic localization. A tool tip window displays exon/intron features of the gene (number, length, start and end positions on the genomic sequence). Two clickable buttons at the top of the entry, namely "Associated Cluster" and "Export sequence", provide the user with a direct access respectively to the associated protein Cluster entry and to the export management tool for sequence extraction.

In some cases, the same entry code is assigned to more than one MitoRes entry that can be distinguished by the presence of a '_' sign followed by a progressive number. This happens when more than one copy of a gene, differing in their number of exons or in their genomic locations, has been annotated for the same protein in the ENSEMBL database. The convention adopted by MitoRes of generating different entries for the same protein for each available gene annotation allows the user to be aware of their existence and to extract only the sequences that are more suitable for their own analysis.

## Utility

Mitochondria-related sequences, despite being available for a large number of metazoan organisms, are dispersed among many heterogeneous resources and this poses a major problem of optimal information extraction. MitoRes tries to fill this gap by collecting and integrating the information on all mitochondrial related sequences in Metazoa from the most accredited worldwide resources and providing a user-friendly web interface through which the user can browse and query the database, extract sequences, compare protein sequence and gene structure among different organisms and perform the alignment of any sequence against the MitoRes collections.

The Web GUI of MitoRes has been built up using a PHP Seagull Framework [20]. BioPerl modules [21] are used to build up the gene and transcript maps and for sequence management in the export tool. A horizontal menu bar, accessible from any MitoRes GUI application, provides links to the MitoRes home page, to the search form, to the Cluster section, to the BLAST search tool and to an extensive on-line users' Manual.

### Search tool

The MitoRes search tool provides several query options and as a result builds up a sortable table of the retrieved entries and of key related available information. Users can query the MitoRes database through the "Quick search" option, the more elaborated search form and/or through the Cluster section.

The "quick search" option in the MitoRes home page takes as input MitoRes ID, UniProt AC and/or gene name and also accepts lists of these search terms

**Figure 2:**
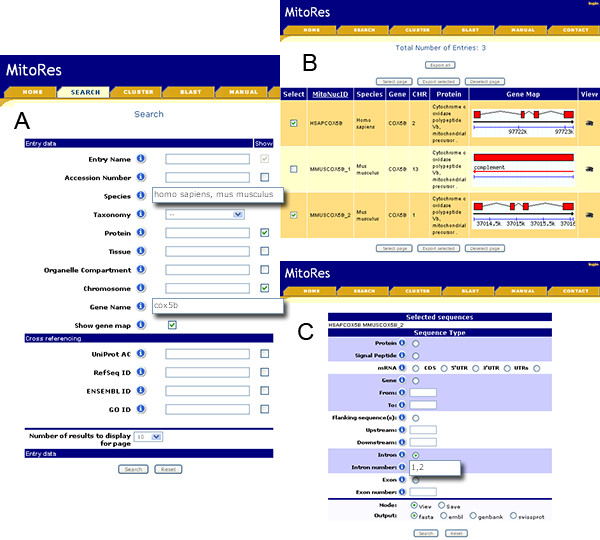
**Database search form, query result page and sequence export form**. A) Search form. The "i" button provides information on how the search field can be used. The "Show" check box provides the possibility to include (checked) or exclude (unchecked) information on the relevant field in the query result page. The search fields "Entry name", "Accession Number", "Species", "Chromosome", "Gene name" and database "Cross-referencing" accept lists of search terms. B) Query result page. Results are displayed as a table summarising information on retrieved entry on the basis of data field chosen in the query form. The "View" button, when pressed, shows the MitoRes entry view. The "Select page"/"Deselect page" buttons allows the selection/deselection of retrieved records for the export of associated sequences. The "Export selected" and "Export all" buttons provide access to the sequence export form for the extraction and export of selected records or of all retrieved records respectively. C) Sequence export form. Selected record/s for sequence export is/are listed at the top. The "i" button provides information on how the extraction of each sequence can be performed. Check boxes at the bottom allow users to choose the mode and sequence file format for export.

The advanced search form (Figure 2A) allows users to browse and search the database through the use of different categories of data. Users can search the database using any of these data categories or different combinations and access MitoRes entries, satisfying the query criteria, from the query results page (Figure 2B).

The query result page presents a sortable list of MitoRes entries matching the query criteria, along with appropriate summary information, set as defaults by the system (organism, gene name, chromosome location and protein description) or chosen by the user during the customization of the query using the "Show" check boxes (see Figure 2A). From the query result page, users can explore the complete contents of each entry and perform the export of associated sequences using the Sequence export form (Figure 2C).

Users can also browse the database using the protein Cluster GUI, where database entries of proteins sharing a certain degree of sequence similarity are grouped in Clusters (see Data sources and implementation paragraph). On this page Clusters are listed in a table that reports the name of the Cluster, the function of the Leader Protein, the list of MitoRes entries belonging to the same Cluster, and provides the link to the Cluster entry.

**Figure 3:**
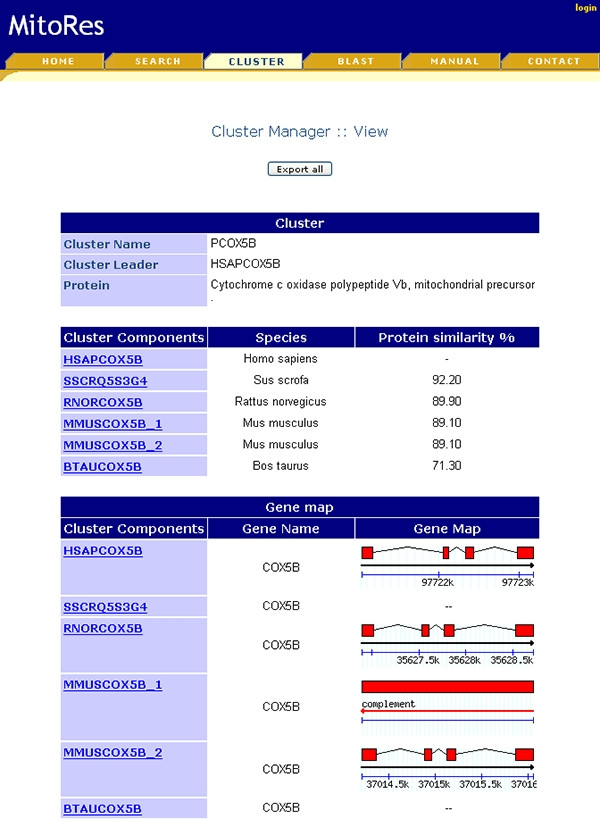
**Cluster entry view**. The Clusters list is accessible clicking the "Cluster" button in the MitoRes home page and/or the "Associated Cluster" button in the MitoRes entries. The "Export all" button at the top, allows the access to the sequence export form to perform the extraction and export of sequences associated to all the Cluster members.

The Cluster entry (Figure 3), in addition to information on the sequence similarity of each protein with respect to the Leader protein, also provides a link to the database entry of Cluster members and direct access to the export management tool ("Export all" buttons) for the extraction of all the protein sequences associated to the Cluster or of any other type of sequences (Figure 2C). Additionally, a "Gene map" section reports the gene name and map for each Cluster member.

A good example of the flexibility and utility of MitoRes is the COX5B gene showed in Figure 2. The query is carried out combining the two search criteria, species and gene name, and using the option for the display of the gene map (Figure 2A) on the query result page (Figure 2B). The utility provided by the system to use several search terms for the same search criterion, allows it to perform the search for the same gene in two different species, namely *H. sapiens *and *M. musculus *in the example described. The query returns 3 matching records. It is immediately evident that two different copies of this gene exist in mouse, one located on chromosome 13 and the other on chromosome 1. The comparison of the gene maps allows the user to quickly asses that the gene structure is extremely conserved in man and mouse and that only one of the two mouse gene copies (MitoRes entry: MMUSCOX5B_2) could be the putative orthologue, the second copy (MitoRes entry: MMUSCOX5B_1), in chromosome 13, probably being derived by a retrotransposition event that should have happened after the divergence of the two organisms or lost in man.

The degree of similarity between the protein sequences can be quickly assessed browsing the COX5B Cluster entry (Figure 3). The information reported in the Cluster entry allows the user to assess that 1) protein annotations are also available for *R. norvegicus*, *S. scrofa *and *B. taurus*, 2) the protein is well conserved in all the organisms for which annotation is available, 3) the degree of similarity with the human protein is, as expected, higher in *S. scrofa *and *R. norvegicus *than in *M. musculus*, 4) genomic annotation is also available for *R. norvegicus *but not for *S. scrofa *and *B. taurus*, 5) the gene structure in *R. norvegicus *also seems identical to that of the other organisms and finally, 6) only *M. musculus *has a COX5B gene copy. At this point users have all the information on COX5B available in MitoRes, and thus can decide which sequences best fit their requirements so bypassing the tedious record-by-record query steps or BLAST database searches, which would otherwise be necessary.

### Sequence export tool

The sequence-export manager tool can run sequence extraction from the entry view page, query results table and Cluster view page. The web view of the sequence export form for performing the extraction of sequence data from searched matching entries is shown in Figure 2C. Depending on the specific user's requirements the system is able to extract: 1) the unprocessed protein sequence and the signal peptide; 2) the complete mRNA sequence, the CDS and the untranslated non coding regions; 3) the complete gene sequence or part of it and the flanking gene regions up to 5000 bp; 4) all the intron and exon sequences or only those specifically chosen by users.

One particularly noteworthy feature of the system is to perform the extraction of gene intron sequences, which is not possible from any other public resource. Furthermore, no limits are imposed regarding the number of sequences which can be extracted and exported. File formats for saving sequence(s) include: FASTA, EMBL, GenBank and SwissProt.

### BLAST tool

The BLAST [22] search tool facilitates the database searching for the functional characterization of unknown sequences. Through this application the user has the option to choose whether to perform the BLAST search against protein, gene or RNA collections of the MitoRes database.

## Availability and requirements

MitoRes is freely available for academic and non-academic users at . User's registration, free of charge, is required only for the use of the BLAST tool. The e_mail bigstaff@ba.itb.cnr.it may be used for comments, suggestions and corrections.

## Authors' contributions

DC performed the data collection and the protein clustering and also participated in the design and implementation of the annotation procedures. FL designed and implemented the relational scheme of the database and the data annotation procedures. AT developed the web GUI interface. GG designed and implemented the data annotation procedures. CS gave her invaluable support as an expert on mitochondria and mitochondrial databases, also providing precious suggestions on the general directions and innovative features of the database. DD coordinated and supervised the whole project, participated in the design of the database and of the web-server interface and drafted the manuscript. All authors read and approved the final manuscript.
